# The geriatric nutrition risk index is longitudinally associated with incident Sarcopenia: evidence from a 5-year prospective cohort

**DOI:** 10.1007/s40520-024-02725-7

**Published:** 2024-03-05

**Authors:** Qiao Xiang, Yuxiao Li, Rui Liang, Quhong Song, Linghui Deng, Birong Dong, Jirong Yue

**Affiliations:** Department of Geriatrics and National Clinical Research Center for Geriatrics, West China Hospital of Sichuan University, 37 GuoXue Lane, Chengdu, Sichuan 610041 P.R. China

**Keywords:** Sarcopenia, GNRI, Prospective cohort study

## Abstract

**Background:**

Previous studies investigating the association between the geriatric nutrition risk index (GNRI) and sarcopenia either lacked longitudinal evidence or narrowly focused on specific populations.

**Aims:**

We aimed to reveal longitudinal associations of GNRI with sarcopenia risk in community-dwelling Chinese. We also investigated interaction effects of potential factors on such associations.

**Methods:**

We included participants aged ≥ 50 years with sufficient data from the WCHAT study who did not have sarcopenia at baseline and completed sarcopenia assessment during follow-up. GNRI was calculated according to the formula based on serum albumin, height and weight. Sarcopenia was diagnosed according to the 2019 AWGS consensus. Longitudinal associations between GNRI and sarcopenia were estimated by logistic regression with GNRI as either a continuous or categorical variable by tertiles, using generalized estimating equations (GEE) as sensitivity analyses. Subgroup analyses by potential covariates were conducted to detect interaction effects.

**Results:**

A total of 1907 participants without baseline sarcopenia were finally included, of whom 327 (17.1%) developed incident sarcopenia during 5-year follow-up. After controlling for confounders, sarcopenia risk decreased with each one standard deviation increase in GNRI (OR_adjusted_=0.36, 95% CI 0.31–0.43), and it also decreased successively from the lowest (< 111.2) through middle (111.2-117.7) to the highest (≥ 117.8) tertile of the GNRI level (P for trend < 0.001). Similar results were yielded by GEE. Such associations generally remained robust across subgroups with distinct characteristics, while significant differences were observed between different age groups (≥ 65 vs. <65 years) (interaction P-value < 0.05).

**Conclusion:**

GNRI is longitudinally associated with sarcopenia risk with possibly age-specific differences in association magnitude, which holds implications for policymakers to conduct population-based risk assessment.

**Supplementary Information:**

The online version contains supplementary material available at 10.1007/s40520-024-02725-7.

## Introduction

Sarcopenia, a degenerative disease characterized by gradual loss of skeletal muscle mass, strength and quality [1, 2], is a common geriatric condition with a prevalence ranging from 10 to 27% in adults [3]. Sarcopenia presents a major public health concern due to various resultant clinical and societal consequences, including reduced quality of life, falls, fractures, frailty, physical limitation, loss of independence, high health care cost and mortality [4–7], attaching significance to its early identification followed by timely management to minimize adverse outcomes and healthcare burden.

According to both the Asian [8] and European [9] consensus, an indispensable dimension for diagnosing sarcopenia is muscle mass, which can be measured using either dual-energy X-ray absorptiometry (DXA) or bioelectrical impedance analysis (BIA). However, these tools may be unavailable for a considerable number of primary care settings with limited medical resources, especially in underdeveloped regions, possibly leading to lack of awareness and recognition towards sarcopenia. One feasible approach can be to develop simpler, cheaper and better popularized predictors, which may early identify high-risk individuals for developing sarcopenia among currently normal ones and allow intervention within the optimal time window.

Malnutrition is closely implicated in the pathogenesis of sarcopenia [10, 11], highlighting potential value of some nutritional status-related indices in predicting sarcopenia risk, including the geriatric nutrition risk index (GNRI). Calculated based on albumin, weight and height, GNRI has been suggested as a cost-effective index to readily and objectively perform nutritional screening [12], although it was originally proposed for prognosis prediction in hospitalized older patients [13]. Previous studies have investigated the association between GNRI and sarcopenia, but so far they either lacked longitudinal evidence to explore causality or narrowly focused on individuals under special conditions [14–22].

In this study, we aimed to provide insights into longitudinal associations of GNRI with risk of sarcopenia in community-based Chinese adults aged ≥ 50 years. We also investigated interaction effects of potential factors on such associations.

## Methods

### Study design and participants

The present study belonged to the West China Health and Aging Trend (WCHAT) study, an ongoing project launched in 2008 and registered on the Chinese Clinical Trial Registry (ChiCTR1800018895). Briefly speaking, community-dwelling participants aged ≥ 50 years were recruited from various areas of west China at baseline (in 2018) based on pre-established eligibility and exclusion criteria, and they were followed up annually either through on-site visits (in 2019, 2021, 2022 and 2023) or by telephone (in 2020). Questionnaire survey, physical examinations and laboratory examinations were conducted for the on-site follow-ups. The project received approval from the Ethical Committee of Sichuan University West China Hospital and adhered to the principles of the Declaration of Helsinki, with written informed consent obtained from all participants or their guardians prior to the project initiation. Additional details about the cohort profile were presented in previous publications [23].

Regarding the analytic sample of this 5-year prospective cohort study, inclusion criteria: (1) participants with sufficient data on height, weight, and serum albumin in 2018; (2) participants who completed sarcopenia assessment in 2018. Exclusion criteria: (1) participants who were confirmed with sarcopenia in 2018; (2) participants who failed to complete sarcopenia assessment at any follow-up point during 2021–2023. Follow-up data for analyses from 2021 onwards were drawn, since only questionnaire-based information obtained by telephone was available in 2020 due to the severe coronavirus disease pandemic.

### Exposure-GNRI

GNRI was calculated according to the formula: GNRI = 1.489 × serum albumin (g/L) + 41.7 × actual weight/ideal weight (kg). The ideal weight was derived from the Lorentz formula as follows: ideal weight for women = 0.60 × height (cm) – 40, ideal weight for men = 0.75 × height (cm) – 62.5, and the actual weight/ ideal weight ratio was set to 1 if the actual weight exceeded the ideal weight [13]. Serum albumin levels were determined using fasting blood samples taken in the early morning. Height and weight were each measured twice by well-experienced staffs with the average value used for analysis.

### Outcome-sarcopenia

The primary outcome of this study was defined as the first, newly diagnosed sarcopenia (incident sarcopenia) among the given follow-up timepoints. As recommended by the 2019 Asian Working Group for Sarcopenia (AWGS) consensus, sarcopenia was diagnosed based on low muscle mass (LMM) plus low muscle strength (LMS) and/or low physical performance (LPP) [8].

LMM was indicated by an appendicular skeletal muscle mass index (ASMI) below 7.0 kg/m^2^ for men and below 5.7 kg/m^2^ for women. ASMI was obtained from the BIA equipment Inbody 770 (BioSpace, Seoul, Korea). LMS was assessed by handgrip strength below 28 kg for men and below 18 kg for women. The dynamometer EH101 (Camry, Zhongshan, China) was used to measure handgrip strength. Subjects stood upright with their feet separated and arms drooping naturally and were asked to grip the dynamometer handle with the dominant hand to their full capacity, and testing was performed on two independent occasions with the largest value recorded for analysis. LPP was identified by a gait speed of < 1.0 m/s in the 6-m walking test or a time of ≥ 12s in the 5-time chair stand test. Measurements of handgrip strength, physical performance and operation of body composition techniques were performed by well-trained and experienced staffs.

### Covariates

BMI was calculated as weight (kg) divided by the square of height (m^2^). Other covariates were derived from questionnaire survey through face-to-face interviews by well-trained medical students or volunteers, including age, sex, ethnicity, marital status, education level, smoking history, alcohol consumption history, activities of daily living (ADL), instrumental ADL (IADL), physical activity level, cognitive function, depression level, sleep quality and number of comorbidities.

ADL or IADL each represents daily self-care activities to support fundamental functioning or independent living [24], with ADL or IADL impairment identified by a total Barthel Index score below 100 or Lawton IADL Scale score below 14, respectively [25, 26]. Physical activity level was determined by the validated China Leisure Time Physical Activity Questionnaire (CLTPAQ) [27], which was a modified version of the Minnesota Leisure Time Physical Activity Questionnaire (MLTPAQ) [28] adapted to Chinese lifestyle and cultural background. As previously detailed, CLTPAQ measured the total amount of energy (kcal) per week spent on a series of commonly performed physical activities, and the sex-specific threshold for low physical activity was the lowest 20th sex-specific percentile value of total energy consumption [29]. Cognitive function was assessed by the Short Portable Mental Status Questionnaire (SPMSQ), with ≥ 5 errors considered as moderate to severe cognitive impairment [30]. Depression level was assessed by the 15-item Geriatric Depression scale (GDS-15), with a score of ≥ 9 indicating moderate to severe or depression [31]. Sleep quality was assessed by the Pittsburgh Sleep Quality Index (PSQI) score, with a score of > 10 considered to be poor [32]. Number of comorbidities referred to the total number of self-reported chronic diseases among hypertension, diabetes, cardiovascular disease, respiratory disease, gastrointestinal disease, hepatic disease, renal disease, neuropsychological disease, stroke and cancer. The confounders to be adjusted for were chosen from among important correlative factors of sarcopenia or GNRI revealed by existing evidence [33–35], providing scientific rationalization for their potential interference with the association between GNRI and sarcopenia. Also, we considered variables with significant baseline differences grouped by tertiles of GNRI levels (see Table 1) and by whether to develop incident sarcopenia during follow-up (see Table S2). Besides, the choice of these covariates took into account balance between complexity and convergence of the models of logistic regression and generalized estimating equations.

### Statistical analysis

Descriptive statistics were presented as mean with standard deviation or median with lower and upper quartile (Q1, Q3) for normally or non-normally distributed continuous variables, and number with percentage (%) for categorical variables. Data comparison between groups was performed by the one-way analysis of variance (ANOVA) or Kruskal-Wallis H test for continuous variables in normal or skewness distribution, and by the Chi-squared test or Fisher’s exact test for categorical variables.

Since the affirmatory occurrence timepoints of sarcopenia were actually unavailable, we assessed longitudinal associations between GNRI and sarcopenia by logistic regression models. To capture the changing trends of sarcopenia at multiple follow-up timepoints with potential coexistence of both progression and reversal [36, 37], we visualized observed transitions between normal and sarcopenia during 2021–2023 by the Sankey diagram using Python’s Plotly library (version 5.4.0). To account for such dynamic nature of sarcopenia and repeated measurements during the follow-up process, we also used the generalized estimating equations (GEE) to assess associations as sensitivity analyses, which can capture the average effects among variables over time. This approach enhances the analytical power by leveraging the augmented number of observations [38, 39]. Estimates by logistic regression and GEE were provided as the odds ratio (OR) and 95% confidence interval (CI) treating GNRI as either a continuous or categorical variable by tertiles, with three models applied. Model 1 was unadjusted for any factors; model 2 was adjusted for age and sex; model 3 was adjusted for age, sex, ethnicity, marital status, education level, smoking history, alcohol consumption history, physical activity level, cognitive function, depressive status, sleep quality, and number of comorbidities. P for trend was calculated for GNRI as a categorical variable. Subgroup analyses were further conducted by potential covariates to detect their interaction effects indicated by interaction P value. Besides, we conducted sensitivity analyses on longitudinal associations between GNRI and sarcopenia through logistic regression, in which sarcopenia was defined according to the revised European consensus on definition and diagnosis (EWGSOP2) [9]. Additionally, we performed sensitivity analyses adjusting for the number of comorbidities as a continuous variable and individual comorbid diseases.

We used Python (version 3.10.10) and R (version 4.3.1) for all statistical analyses. P values < 0.05 were considered statistically significant.

## Results

**Fig. 1:**
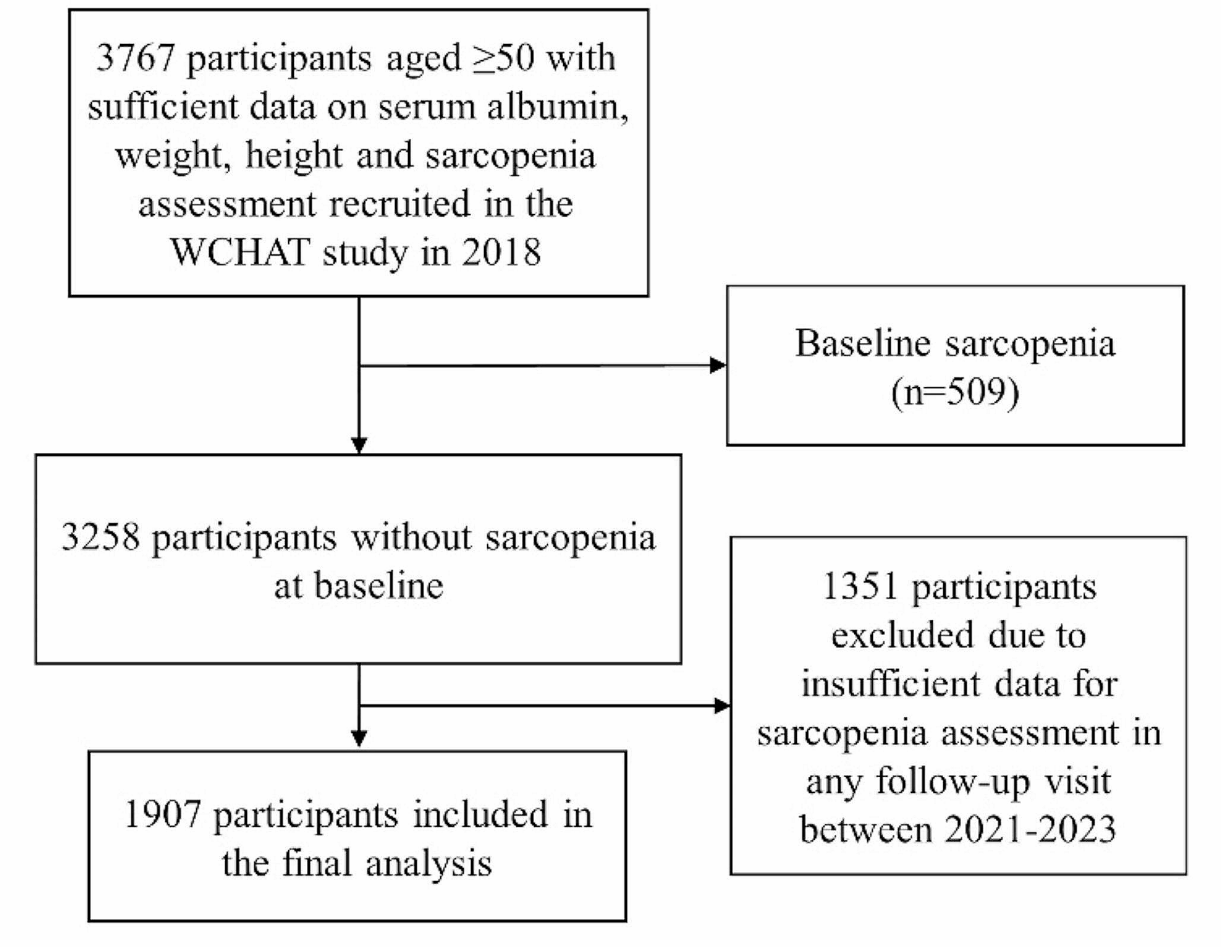
The flowchart of inclusion and exclusion of participants. **Abbreviations**: **WCHAT**, West China Health and Aging Trend

A total of 3767 participants with complete data for the exposure and outcome at baseline were initially enrolled. Then we excluded those having sarcopenia at baseline (*n* = 509), and those with insufficient data for sarcopenia assessment at any follow-up point (*n* = 1351), leaving 1907 participants in the final analytic sample (Fig. 1). We divided them into three groups by tertiles of their baseline GNRI levels: tertile 1 (T1 or low, < 111.2), tertile 2 (T2 or middle, 111.2-117.7), tertile 3 (T3 or high, ≥ 117.8). Baseline characteristics between participants excluded due to insufficient information and those included in the analytic sample were presented in Table S1.

At study entry, participants with the lowest GNRI levels (T1) were more likely to be older, men, have smoking history, physically inactive, have lower levels of albumin, BMI and ASMI, while they were less likely to have multicomorbidity, compared with the other two groups (T2 and T3) (Table 1).

**Table 1:** Baseline characteristics of the 1907 participants in the analytic sample by tertiles of GNRI levels

	Tertile 1 *n* = 636	Tertile 2 *n* = 635	Tertile 3 *n* = 636	P Value
**Age**	62.29 (7.78)	61.58 (7.33)	60.89 (7.00)	0.003
**Sex, n (%)**				0.025
Male	223 (35.06)	201 (31.65)	178 (27.99)	
Female	413 (64.94)	434 (68.35)	458 (72.01)	
**Ethnicity, n (%)**				0.171
Han	165 (25.94)	164 (25.83)	198 (31.13)	
Qiang	210 (33.02)	220 (34.65)	207 (32.55)	
Tibetan	161 (25.31)	159 (25.04)	152 (23.90)	
Yi	79 (12.42)	73 (11.50)	53 (8.33)	
Other minorities	21 (3.30)	19 (2.99)	26 (4.09)	
**Marital status: married, n (%)**	541 (85.06)	554 (87.24)	545 (85.69)	0.514
**Education: high school or above, n (%)**	100 (15.72)	92 (14.49)	79 (12.42)	0.234
**Smoking history, n (%)**	106 (16.67)	66 (10.39)	49 (7.70)	< 0.001
**Alcohol consumption history, n (%)**	152 (23.90)	136 (21.42)	144 (22.64)	0.572
**Low physical activity, n (%)**	172 (27.04)	123 (19.37)	131 (20.60)	0.002
**ADL impairment, n (%)**	56 (8.81)	50 (7.87)	53 (8.33)	0.835
**IADL impairment, n (%)**	108 (16.98)	108 (17.01)	113 (17.77)	0.915
**Moderate to severe cognitive impairment, n (%)**	68 (10.69)	54 (8.50)	69 (10.85)	0.298
**Moderate to severe depression, n (%)**	34 (5.35)	21 (3.31)	30 (4.72)	0.197
**Poor sleep quality, n (%)**	94 (14.80)	73 (11.51)	70 (11.01)	0.084
**Number of comorbidities, n (%)**				0.025
< 2	583 (91.67)	565 (88.98)	553 (86.95)	
>=2	53 (8.33)	70 (11.02)	83 (13.05)	
**Albumin (g/L)**	42.78 (2.44)	44.69 (2.06)	46.28 (2.63)	< 0.001
**BMI (kg/m** ^**2**^ **)**	22.62 (2.14)	25.53 (1.78)	28.82 (2.58)	< 0.001
**ASMI (kg/m** ^**2**^ **)**	6.37 (0.77)	6.70 (0.80)	7.15 (0.82)	< 0.001
**Handgrip strength (kg)**	23.00 (8.38)	23.00 (8.44)	23.84 (8.73)	0.129
**Time consumed in the 6-meter walking test (s)**	4.84 (1.54)	4.87 (1.58)	4.81 (1.45)	0.812
**Time consumed in the 5-time chair stand test (s)**	10.90 (2.66)	10.98 (2.74)	11.02 (2.71)	0.715

*Note*: data were presented as mean (standard deviation) or n (%) as appropriate (continuous variables here were all in normal distribution). P value indicated the significance level for comparison between groups

Tertiles of GNRI: tertile 1, < 111.2; tertile 2, 111.2-117.7; tertile 3, ≥ 117.8

**Abbreviations**: **GNRI**, geriatric nutrition risk index; **ADL**, Activities of Daily Living; **IADL**, Instrumental ADL; **BMI**, body mass index; **ASMI**, appendicular skeletal muscle mass index

During 5-year follow-up, 327 (17.1%) participants developed incident sarcopenia, and at baseline they were more likely to be older, physically inactive, have smoking history, have lower levels of albumin, GNRI, BMI, ASMI, handgrip strength and physical performance, while they were less likely to be married compared with those who remained normal during follow-up (Table S2). For sarcopenia defined by the EWGSOP2^9^, baseline characteristics of participants without sarcopenia at entry grouped by developing incident sarcopenia and remaining normal during follow-up were presented in Table S3.

As suggested by logistic regression, when treated as a continuous variable, a higher GNRI level was associated with lower sarcopenia risk both in the unadjusted and adjusted models (OR_adjusted_=0.36, 95% CI 0.31–0.43). When we treated GNRI as a categorical variable, the risk of sarcopenia increased successively from the T3 through T2 to T1 group (P for trend < 0.001) in all the three models (Table 2). These findings were replicated when we used the EWGSOP2^9^ consensus to define sarcopenia (Table S4) and when we adjusted for the number of comorbidities as a continuous variable or individual comorbid diseases (Table S5).

**Table 2:** Longitudinal associations of GNRI with sarcopenia through logistic regression in different models

	OR (95%CI), P value			P for trend^c^
	per SD increase^a^	Tertile 1^b^ (< 111.2)	Tertile 2^b^ (111.2-117.7)	
**Model 1** ^**d**^	**0.36 (0.31–0.42), < 0.001**	**12.86 (8.22–20.13), < 0.001**	**4.80 (3.01–7.68), < 0.001**	**< 0.001**
**Model 2** ^**e**^	**0.36 (0.30–0.42), < 0.001**	**12.71 (8.06–20.02), < 0.001**	**4.72 (2.94–7.59), < 0.001**	**< 0.001**
**Model 3** ^**f**^	**0.36 (0.31–0.43), < 0.001**	**12.27 (7.73–19.47), < 0.001**	**4.79 (2.97–7.73), < 0.001**	**< 0.001**

*Note*: a. Estimates were provided with per standard deviation increase in the GNRI level

b. Estimates were provided with GNRI ≥ 117.8 (tertile 3) as reference

c. P for trend was calculated for GNRI as a categorical variable by tertiles

d. Model 1 was unadjusted for any factors

e. Model 2 was adjusted for age and sex

f. Model 3 was adjusted for age, sex (male vs. female), ethnicity (non-Han vs. Han Chinese), marital status (single, divorced or widowed vs. married), education level (high school or above vs. middle school or lower), smoking history (yes vs. no), alcohol consumption history (yes vs. no), physical activity level (low vs. normal), cognitive function (moderate to severe impairment vs. normal), depression level (moderate to severe vs. normal), sleep quality (low vs. normal), and number of comorbidities (≥ 2 vs. <2)

**Abbreviations: GNRI**, geriatric nutrition risk index; **OR**, odds ratio; **CI**, confidence interval; **SD**, standard deviation

Considering the dynamic nature of sarcopenia depicted by inflows and outflows of status at multiple follow-up timepoints during 2021–2023 (Figure S1), sensitivity analyses by GEE were additionally performed, which also revealed that the GNRI level was negatively associated with sarcopenia risk both in the unadjusted and adjusted models (OR_adjusted_=0.37, 95% CI 0.31–0.43). Similar results were yielded when we treated GNRI as a categorical variable (P for trend < 0.001 in all the three models) (Table 3).

**Table 3:** Longitudinal associations of GNRI with sarcopenia through GEE in different models

	OR (95%CI), P value			P for trend^c^
	per SD increase^a^	Tertile 1^b^	Tertile 2^b^	
**Model 1** ^**d**^	**0.36 (0.31–0.42), < 0.001**	**11.82 (7.29–19.17), < 0.001**	**4.78 (2.88–7.95), < 0.001**	**< 0.001**
**Model 2** ^**e**^	**0.36 (0.31–0.42), < 0.001**	**11.45 (6.99–18.76), < 0.001**	**4.54 (2.72–7.59), < 0.001**	**< 0.001**
**Model 3** ^**f**^	**0.37 (0.31–0.43), < 0.001**	**11.08 (6.71–18.29), < 0.001**	**4.60 (2.75–7.69), < 0.001**	**< 0.001**

*Note*: GEE models were fitted using the exchangeable correlation structure with robust estimation of the standard errors. The binomial response was selected for the distribution and link function

a. Estimates were provided with per standard deviation increase in the GNRI level

b. Estimates were provided with GNRI ≥ 117.8 (tertile 3) as reference

c. P for trend was calculated for GNRI as a categorical variable by tertiles

d. Model 1 was unadjusted for any factors

e. Model 2 was adjusted for age and sex

f. Model 3 was adjusted for age, sex (male vs. female), ethnicity (non-Han vs. Han Chinese), marital status (single, divorced or widowed vs. married), education level (high school or above vs. middle school or lower), smoking history (yes vs. no), alcohol consumption history (yes vs. no), physical activity level (low vs. normal), cognitive function (moderate to severe impairment vs. normal), depression level (moderate to severe vs. normal), sleep quality (low vs. normal), and number of comorbidities (≥ 2 vs. <2)

**Abbreviations: GNRI**, geriatric nutrition risk index; **GEE**, generalized estimating equations; **OR**, odds ratio; **CI**, confidence interval; **SD**, standard deviation

**Fig. 2:**
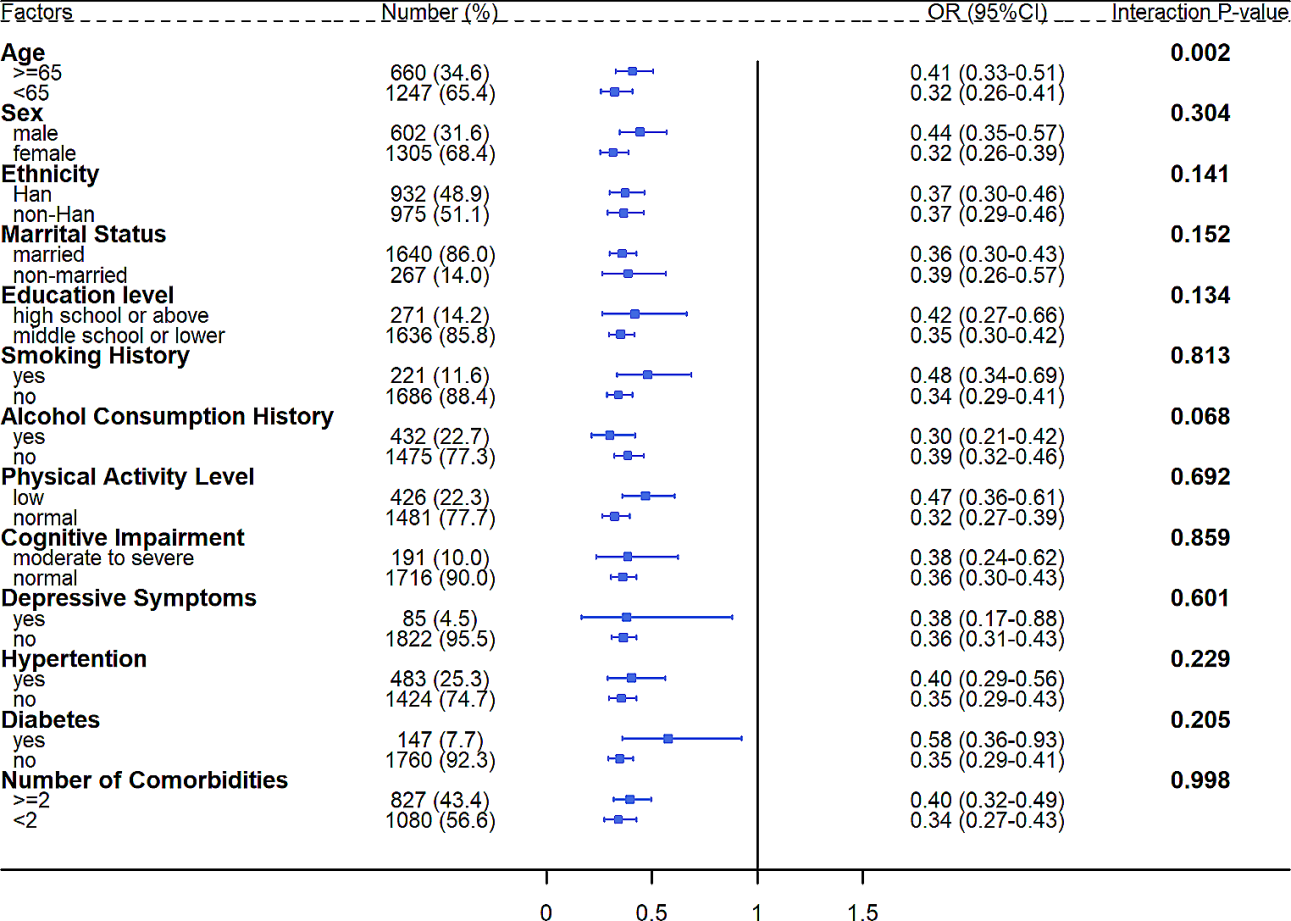
Subgroup analyses to detect interaction effect between GNRI and the concerned confounders on sarcopenia. *Note*: For each confounder (except hypertension and diabetes) investigated in subgroup analyses, estimates were obtained based on model 3 but without adjustment for the relevant confounder. For hypertension and diabetes, estimates were obtained based on model 3 but without adjustment for number of comorbidities. Model 3 was adjusted for age, sex (male vs. female), ethnicity (non-Han vs. Han Chinese), marital status (single, divorced or widowed vs. married), education level (high school or above vs. middle school or lower), smoking history (yes vs. no), alcohol consumption history (yes vs. no), physical activity level (low vs. normal), cognitive function (moderate to severe impairment vs. normal), depression level (moderate to severe vs. normal), sleep quality (low vs. normal), and number of comorbidities (≥ 2 vs. <2). **Abbreviations: OR**, odds ratio; **CI**, confidence interval

As indicated by subgroup analyses, the impact of GNRI on sarcopenia varied across different age groups (OR_adjusted_=0.41, 95% CI 0.33–0.51 for ≥ 65 years; OR_adjusted_=0.32, 95% CI 0.26–0.41 for < 65 years; interaction P-value = 0.002), while no significant interaction effect was observed between GNRI and the other concerned confounders on sarcopenia (interaction P-value > 0.05) (Fig. 2).

## Discussion

In this study, we revealed negative associations between the GNRI level and sarcopenia risk even after controlling for the confounders, and such associations generally remained robust across subgroups with distinct characteristics, while significant differences were observed between different age groups.

Consistent with our findings, previous cross-sectional studies [14–22] have demonstrated correlations of GNRI with sarcopenia, which served as the foundation of our current research and motivated us to further unravel the longitudinal associations. In some studies conducted in Asian population [14, 17, 18], sarcopenia diagnosis was only based on LMM plus LMS but did not consider LPP due to lack of relevant data. There is ongoing controversy regarding the interpretation of LPP in sarcopenia assessment. Although physical performance was not a necessary criterion for sarcopenia diagnosis but used for severity classification according to the European consensus [9], it was still retained as one of the diagnostic dimensions for sarcopenia by AWGS [8], because it is not only among the strongest predictors of geriatric health outcomes [40, 41], but also easy to measure in primary care without relying on special equipment unlike muscle mass or strength [42]. Consideration of LPP in our study may thus reduce the likelihood of misclassifying some sarcopenic patients as normal, thus strengthening validity of results. Besides, associations between GNRI and sarcopenia in many previous studies were established in specific populations, including patients with cirrhosis [14], type 2 diabetes mellitus (T2DM) [16–18], maintenance hemodialysis [19] and malignancies [20–22], which may limit generalizability of the findings. Although emphasis should be attached to sarcopenia in these special populations considering its prognostic implications for the diseases [43–46], its early identification also deserves attention in community-dwelling older adults, since it is predictive for mortality in this population [47, 48], which may show more significance from a public health perspective. Therefore, our findings derived from community-based settings can supplement existing evidence. For example, as suggested by our subgroup analyses, the longitudinal relationship between GNRI and sarcopenia was not moderated by diabetes history, further supporting that previous evidence restricted to T2DM population [16–18] may be extrapolated regardless of T2DM history.

The longitudinal association of GNRI with sarcopenia risk may be explained by the role malnutrition and systemic chronic inflammation play in sarcopenia pathology [49, 50], both of which can be reflected by the GNRI level [12, 51]. On the one hand, malnutrition is a driving factor of sarcopenia, because it is accompanied by decreased protein synthesis and increased protein degradation, leading to subsequent loss of muscle mass, strength and quality [49]. GNRI shows advantages in assessing nutritional status. Some subjective, questionnaire-based tools, such as the Short-Form Mini Nutritional Assessment [52], may not apply to older people with cognitive impairment or communication difficulties, while GNRI is a simpler and more objective measure. Also, GNRI may outperform some anthropometric indices such as the calf circumference or mid-arm circumference, which are more prone to interpretation errors since adipose or connective tissues and edema can take the place of muscle tissues [53]. Notably, GNRI takes into account albumin, weight and height simultaneously, which may help minimize confounders such as hydration status compared with albumin alone [13, 54]. On the other hand, elevated proinflammatory cytokines can induce proteolysis and contribute to sarcopenia by activating the ubiquitin-proteasome system [50], while GNRI has been reported to correlated with inflammation indicators [51]. Exact biophysiological mechanisms of the relationship between GNRI and sarcopenia remain to be clarified in future research.

Besides, our findings were generally robust across subgroups, except for age potentially being a moderator in the relationship between GNRI and sarcopenia risk. Although the longitudinal association was observed in both of the age subgroups, it was even stronger for people aged < 65 years. This implies that enhancing nutrition and improving chronic inflammatory status may be even more protective against sarcopenia from a younger age, since differences in the pace of aging are evident early in adulthood [55]. Regarding possible explanations, we hypothesized that as people age, improving malnutrition and inflammation may result in less evident benefits for muscle mass and function, possibly due to factors such as hormonal changes, decreased physical activity, or the presence of other age-related diseases, with the underlying mechanisms remaining to be elucidated.

Our study supports the utility of GNRI to identify people at high risk for sarcopenia among currently normal ones and to shed light on the time window when intervention should be enhanced to prevent sarcopenia. This may add practical value to primary healthcare practice, which can be particularly applicable to situations resembling our research participants and settings. Our study population is from the underdeveloped western region of China with comparatively poor medical conditions and limited resources, where techniques such as DXA, BIA or even the dynamometer are not widely available. Besides, most older residents there have low education levels and weak health awareness, and what they are most concerned about are still the well-known health issues such as hypertension, diabetes, and cancer rather than sarcopenia alone. They do not have a strong desire to monitor and manage sarcopenia due to concerns about cost or radiation exposure and lack of knowledge about sarcopenia. On the flip side, physicians in primary care there have a demanding workload and even they rarely focus on the issue of sarcopenia. Thus, it is unrealistic to carry out a standardized sarcopenia assessment regularly in the large cardinal number of population. Nevertheless, by measuring GNRI, which is cost-effective and accessible from routine laboratory tests and anthropometric measurements, we can monitor muscle conditions incidentally along with other common health issues in a simple way. This is much more acceptable for the local population and may not additionally add to physicians’ burden.

To our knowledge, this is the first longitudinal study to unveil the association between GNRI and sarcopenia based on community-dwelling Chinese throughout a 5-year observation. It overcame previous limitation of cross-section designs or studying only specific populations, and as discussed above, it may serve as a supplement for existing evidence focused on participants aged ≥ 50 years in less-developed areas, which may hold significant implications for policymakers to conduct population-based risk assessment.

Despite the strengths, some limitations should also be recognized. First, exclusion of participants with unavailable information necessary for analysis might introduce potential selection bias. Indeed, the excluded participants showed generally poorer health status than the included ones at baseline (Table S1). A possible explanation was that those experiencing more physical or functional limitations reasonably seemed less likely to attend the follow-up center and complete data collection. Therefore, our findings may be more conservative estimates which should be treated with caution. Regardless of the differences, the included sample still aligns with the general population prone to sarcopenia regarding diversity in sociodemographic, lifestyle-related and health-related characteristics. Second, despite our efforts to control for an adequate number of confounders to minimize their interference with result interpretation, there might still be other residual factors that have not been considered. Third, participants recruited in the WCHAT were comparatively younger ones (aged ≥ 50 year) residing in western China, which suggests the need for caution in result extrapolation. Factors like ethnicity, lifestyles, educational and socioeconomic levels can vary significantly across populations, and there are different criteria, measurement methods or cut-off points for sarcopenia assessment. Therefore, our findings require validation in older populations of other regions or even countries. However, considering the progressive decline in muscle mass and strength after the fourth decade of life, sarcopenia can occur at an early age, and therefore our findings focused on adults aged ≥ 50 years are valuable for such younger population whose sarcopenia identification and prevention is receiving increasing attention. Fourth, disease information obtained through self-reporting might introduce the possibility of misclassification, and more objective measures such as linkage to medical records are expected in future studies for disease verification. Moreover, currently we have no access to an independent sample to validate the predictive value of GNRI through the receiver operating characteristic analysis, which can be further investigated in later research.

In conclusion, GNRI is longitudinally associated with sarcopenia risk with possibly age-specific differences in association magnitude. By measuring GNRI at an observation, we can assess relative chances of developing sarcopenia though referring to the high-level category; we can also dynamically observe GNRI trends through follow-up to track changes in muscle conditions and monitor intervention effect. Further evidence from prospective cohorts with larger sample size, longer observation length and more representative population is needed to validate our findings.

### Electronic supplementary material

Below is the link to the electronic supplementary material.

Supplementary Material 1
