# Supplementary material for: The geriatric nutrition risk index is longitudinally associated with incident Sarcopenia: evidence from a 5-year prospective cohort
Source: Aging Clin Exp Res. 2024 Mar 5;36(1):52. doi: 10.1007/s40520-024-02725-7 (PMC10912133; doi:10.1007/s40520-024-02725-7)
Supplement: Supplementary file 1 — Supplementary Material 1 [file 40520_2024_2725_MOESM1_ESM.docx]

Supplementary Material

[Table S1. 2](#_Toc156465374)

[Table S2. 3](#_Toc156465375)

[Table S3. 4](#_Toc156465376)

[Table S4. 6](#_Toc156465377)

[Table S5 7](#_Toc156465378)

[Figure S1. 8](#_Toc156465379)

Table S1. Comparison of baseline characteristics between the participants excluded due to insufficient information and those included in the analytical sample.

|  | **Excluded participants n=1860** | **Included participants n=1907** | **P Value** |
| --- | --- | --- | --- |
| **Age** | 62.97 (8.93) | 61.59 (7.39) | <0.001 |
| **Sex, n (%)** |  |  | <0.001 |
| Male | 745 (40.05) | 602 (31.57) |  |
| Female | 1115 (59.95) | 1305 (68.43) |  |
| **Ethnicity, n (%)** |  |  | <0.001 |
| Han | 708 (38.06) | 932 (48.87) |  |
| Qiang | 301 (16.18) | 669 (35.08) |  |
| Tibetan | 682 (36.67) | 229 (12.01) |  |
| Yi | 133 (7.15) | 53 (2.78) |  |
| Other minorities | 36 (1.94) | 24 (1.26) |  |
| **Marital status: married, n (%)** | 1527 (82.10) | 1640 (86.00) | 0.001 |
| **Education: high school or above, n (%)** | 266 (14.30) | 271 (14.21) | 0.974 |
| **Smoking history, n (%)** | 272 (14.62) | 221 (11.59) | 0.007 |
| **Alcohol consumption history, n (%)** | 339 (18.23) | 432 (22.65) | 0.001 |
| **Low physical activity, n (%)** | 688 (36.99) | 426 (22.34) | <0.001 |
| **ADL impairment, n (%)** | 218 (11.72) | 159 (8.34) | 0.001 |
| **IADL impairment, n (%)** | 486 (26.13) | 329 (17.25) | <0.001 |
| **Moderate to severe cognitive impairment, n (%)** | 323 (17.37) | 191 (10.02) | <0.001 |
| **Moderate to severe depression, n (%)** | 90 (4.84) | 85 (4.46) | 0.632 |
| **Poor sleep quality, n (%)** | 206 (11.09) | 237 (12.44) | 0.216 |
| **Number of comorbidities, n (%)** |  |  | 0.321 |
| < 2 | 1639 (88.12) | 1701 (89.20) |  |
| ≥2 | 221 (11.88) | 206 (10.80) |  |
| **Albumin (g/L)** | 43.87 (3.24) | 44.58 (2.79) | <0.001 |
| **GNRI** | 112.02 (9.43) | 114.53 (7.95) | <0.001 |
| **BMI (kg/m^2^)** | 24.91 (4.03) | 25.66 (3.35) | <0.001 |
| **ASMI (kg/m^2^)** | 6.52 (1.00) | 6.74 (0.86) | <0.001 |
| **Handgrip strength (kg)** | 20.98 (8.55) | 23.28 (8.52) | <0.001 |
| **Time consumed in the 6-meter walking test (s)** | 5.43 (2.59) | 4.84 (1.52) | <0.001 |
| **Time consumed in the 5-time chair stand test (s)** | 12.17 (3.69) | 10.96 (2.70) | <0.001 |

**Note**: data were presented as mean (standard deviation) or n (%) as appropriate (continuous variables here were all in normal distribution). P value indicated the significance level for comparison between groups.

**Abbreviations**: **ADL**, Activities of Daily Living; **IADL,** Instrumental ADL; **GNRI**, geriatric nutrition risk index; **BMI**, body mass index; **ASMI**, appendicular skeletal muscle mass index.

Table S2. Baseline characteristics of the 1907 participants in the analytic sample without sarcopenia at entry grouped by developing incident sarcopenia and remaining normal during follow-up.

|  | **Normal n=1580** | **Sarcopenia n=327** | **P Value** |
| --- | --- | --- | --- |
| **Age** | 60.79 (7.10) | 65.46 (7.58) | <0.001 |
| **Sex, n (%)** |  |  | 0.342 |
| Male | 491 (31.08) | 111 (33.94) |  |
| Female | 1089 (68.92) | 216 (66.06) |  |
| **Ethnicity, n (%)** |  |  | <0.001 |
| Han | 743 (47.03) | 189 (57.80) |  |
| Qiang | 575 (36.39) | 94 (28.75) |  |
| Tibetan | 203 (12.85) | 26 (7.95) |  |
| Yi | 38 (2.41) | 15 (4.59) |  |
| Other minorities | 21 (1.33) | 3 (0.92) |  |
| **Marital status: married, n (%)** | 1378 (87.22) | 262 (80.12) | 0.001 |
| **Education: high school or above, n (%)** | 229 (14.49) | 42 (12.84) | 0.490 |
| **Smoking history, n (%)** | 166 (10.51) | 55 (16.82) | 0.002 |
| **Alcohol consumption history, n (%)** | 349 (22.09) | 83 (25.38) | 0.221 |
| **Low physical activity, n (%)** | 336 (21.27) | 90 (27.52) | 0.016 |
| **ADL impairment, n (%)** | 132 (8.35) | 27 (8.26) | 1.000 |
| **IADL impairment, n (%)** | 263 (16.65) | 66 (20.18) | 0.144 |
| **Moderate to severe cognitive impairment, n (%)** | 153 (9.68) | 38 (11.62) | 0.337 |
| **Moderate to severe depression, n (%)** | 70 (4.43) | 15 (4.59) | 1.000 |
| **Poor sleep quality, n (%)** | 198 (12.54) | 39 (11.96) | 0.845 |
| **Number of comorbidities, n (%)** |  |  | 0.227 |
| < 2 | 1416 (89.62) | 285 (87.16) |  |
| ≥2 | 164 (10.38) | 42 (12.84) |  |
| **Albumin (g/L)** | 44.75 (2.75) | 43.76 (2.80) | <0.001 |
| **GNRI** | 115.74 (7.59) | 108.69 (7.03) | <0.001 |
| **BMI (kg/m^2^)** | 26.16 (3.26) | 23.24 (2.67) | <0.001 |
| **ASMI (kg/m^2^)** | 6.88 (0.82) | 6.08 (0.71) | <0.001 |
| **Handgrip strength (kg)** | 23.81 (8.63) | 20.75 (7.49) | <0.001 |
| **Time consumed in the 6-meter walking test (s)** | 4.81 (1.54) | 4.99 (1.43) | 0.036 |
| **Time consumed in the 5-time chair stand test (s)** | 10.92 (2.73) | 11.15 (2.56) | 0.146 |

**Note**: data were presented as mean (standard deviation) or n (%) as appropriate (continuous variables here were all in normal distribution). P value indicated the significance level for comparison between groups.

**Abbreviations**: **ADL**, Activities of Daily Living; **IADL,** Instrumental ADL; **GNRI**, geriatric nutrition risk index; **BMI**, body mass index; **ASMI**, appendicular skeletal muscle mass index.

Table S3. Baseline characteristics of the 1938 participants in the analytic sample without sarcopenia at entry grouped by developing incident sarcopenia and remaining normal during follow-up (based on the EWGSOP2).

|  | **Normal n=1800** | **Sarcopenia n=138** | **P Value** |
| --- | --- | --- | --- |
| **Age** | 61.18 (7.20) | 67.35 (7.73) | <0.001 |
| **Sex, n (%)** |  |  | <0.001 |
| Male | 544 (30.22) | 75 (54.35) |  |
| Female | 1256 (69.78) | 63 (45.65) |  |
| **Ethnicity, n (%)** |  |  | <0.001 |
| Han | 869 (48.28) | 80 (57.97) |  |
| Qiang | 639 (35.50) | 33 (23.91) |  |
| Tibetan | 227 (12.61) | 8 (5.80) |  |
| Yi | 41 (2.28) | 15 (10.87) |  |
| Other minorities | 24 (1.33) | 2 (1.45) |  |
| **Marital status: married, n (%)** | 1551 (86.17) | 115 (83.33) | 0.426 |
| **Education: high school or above, n (%)** | 261 (14.50) | 17 (12.32) | 0.563 |
| **Smoking history, n (%)** | 194 (10.78) | 35 (25.36) | <0.001 |
| **Alcohol consumption history, n (%)** | 402 (22.33) | 39 (28.26) | 0.135 |
| **Low physical activity, n (%)** | 394 (21.89) | 44 (31.88) | 0.009 |
| **ADL impairment, n (%)** | 149 (8.28) | 15 (10.87) | 0.370 |
| **IADL impairment, n (%)** | 294 (16.33) | 38 (27.54) | 0.001 |
| **Moderate to severe cognitive impairment, n(%)** | 172 (9.56) | 22 (15.94) | 0.024 |
| **Moderate to severe depression, n (%)** | 78 (4.33) | 5 (3.62) | 0.858 |
| **Poor sleep quality, n (%)** | 231 (12.84) | 14 (10.22) | 0.449 |
| **Number of comorbidities, n (%)** |  |  | 0.708 |
| < 2 | 1605 (89.17) | 125 (90.58) |  |
| ≥2 | 195 (10.83) | 13 (9.42) |  |
| **Albumin (g/L)** | 44.66 (2.75) | 43.44 (3.01) | <0.001 |
| **GNRI** | 114.90 (7.83) | 106.90 (7.75) | <0.001 |
| **BMI (kg/m^2^)** | 25.78 (3.36) | 22.64 (2.71) | <0.001 |
| **ASMI (kg/m^2^)** | 6.76 (0.86) | 6.20 (0.88) | <0.001 |
| **Handgrip strength (kg)** | 23.40 (8.54) | 22.95 (7.63) | 0.508 |
| **Time consumed in the 6-meter walking test (s)** | 4.82 (1.53) | 5.06 (1.20) | 0.029 |
| **Time consumed in the 5-time chair stand test (s)** | 10.95 (2.71) | 11.35 (2.36) | 0.063 |

**Note**: Sarcopenia was defined according to the EWGSOP2 criteria: low muscle strength (<27 kg for men and <16 kg for women) plus low muscle mass (< 7.0kg/m2 for men and 6.0 kg/m2 for women).

Data were presented as mean (standard deviation) or n (%) as appropriate (continuous variables here were all in normal distribution). P value indicated the significance level for comparison between groups.

**Abbreviations**: **EWGSOP2,** the revised European consensus on definition and diagnosis; **ADL**, Activities of Daily Living; **IADL,** Instrumental ADL; **GNRI**, geriatric nutrition risk index; **BMI**, body mass index; **ASMI**, appendicular skeletal muscle mass index.

Table S4. Longitudinal associations of GNRI with sarcopenia defined by the EWGSOP2 consensus through logistic regression in different models.

|  | **OR (95%CI), P value** | | | **P for trend^c^** |
| --- | --- | --- | --- | --- |
|  | **per SD increase**^a^ | **Tertile 1**^b^ **(<111.1)** | **Tertile 2**^b^ **(111.1-117.6)** |  |
| **Model 1^d^** | **0.36 (0.29-0.43), <0.001** | **10.83 (5.59-20.99), <0.001** | **3.53 (1.73-7.21), <0.001** | **<0.001** |
| **Model 2^e^** | **0.39 (0.32-0.47), <0.001** | **9.50 (4.86-18.58), <0.001** | **3.21 (1.56-6.62), 0.002** | **<0.001** |
| **Model 3^f^** | **0.40 (0.33-0.50), <0.001** | **8.66 (4.38-17.12), <0.001** | **3.20 (1.55-6.63), 0.002** | **<0.001** |

**Note:** a. Estimates were provided with per standard deviation increase in the GNRI level.

b. Estimates were provided with GNRI ≥ 117.7 (tertile 3) as reference.

c. P for trend was calculated for GNRI as a categorical variable by tertiles.

d. Model 1 was unadjusted for any factors.

e. Model 2 was adjusted for age and sex.

f. Model 3 was adjusted for age, sex (male vs. female), ethnicity (non-Han vs. Han Chinese), marital status (single, divorced or widowed vs. married), education level (high school or above vs. middle school or lower), smoking history (yes vs. no), alcohol consumption history (yes vs. no), physical activity level (low vs. normal), cognitive function (moderate to severe impairment vs. normal), depression level (moderate to severe vs. normal), sleep quality (low vs. normal), and number of comorbidities (≥ 2 vs. <2).

**Abbreviations:** **GNRI**, geriatric nutrition risk index; **EWGSOP2,** the revised European consensus on definition and diagnosis; **OR**, odds ratio; **CI**, confidence interval; **SD**, standard deviation.

Table S5**.** Longitudinal associations of GNRI with sarcopenia through logistic regression adjusting for the number of comorbidities as a continuous variable or individual comorbid diseases in different models.

|  | **OR (95%CI), P value** | | | **P for trend^c^** |
| --- | --- | --- | --- | --- |
|  | **per SD increase^a^** | **Tertile 1^b^ (<111.2)** | **Tertile 2^b^ (111.2-117.7)** |  |
| **Model 1^d^** | **0.37 (0.32-0.43), <0.001** | **11.92 (7.53-18.88), <0.001** | **4.72 (2.93-7.60), <0.001** | **<0.001** |
| **Model 2^e^** | **0.36 (0.31-0.43), <0.001** | **12.32 (7.74-19.60), <0.001** | **4.83 (2.99-7.80), <0.001** | **<0.001** |
| **Model 3^f^** | **0.37 (0.31-0.43), <0.001** | **12.02 (7.52-19.21), <0.001** | **4.78 (2.95-7.75), <0.001** | **<0.001** |

**Note:** a. Estimates were provided with per standard deviation increase in the GNRI level.

b. Estimates were provided with GNRI ≥ 117.8 (tertile 3) as reference.

c. P for trend was calculated for GNRI as a categorical variable by tertiles.

d. Model 1 was adjusted for age, sex (male vs. female), ethnicity (non-Han vs. Han Chinese), marital status (single, divorced or widowed vs. married), education level (high school or above vs. middle school or lower), smoking history (yes vs. no), alcohol consumption history (yes vs. no), physical activity level (low vs. normal), cognitive function (moderate to severe impairment vs. normal), depression level (moderate to severe vs. normal) and sleep quality (low vs. normal).

e. Model 2 was further adjusted for the number of comorbidities as a continuous variable on the basis of model 1.

f. Model 3 was further adjusted for individual comorbid diseases (including hypertension, diabetes, cardiovascular disease, respiratory disease, gastrointestinal disease and stroke) on the basis of model 1. Other comorbid diseases such as cancer were not included in the adjustment of model 3 due to few cases.

**Abbreviations:** **GNRI**, geriatric nutrition risk index; **OR**, odds ratio; **CI**, confidence interval; **SD**, standard deviation.

**
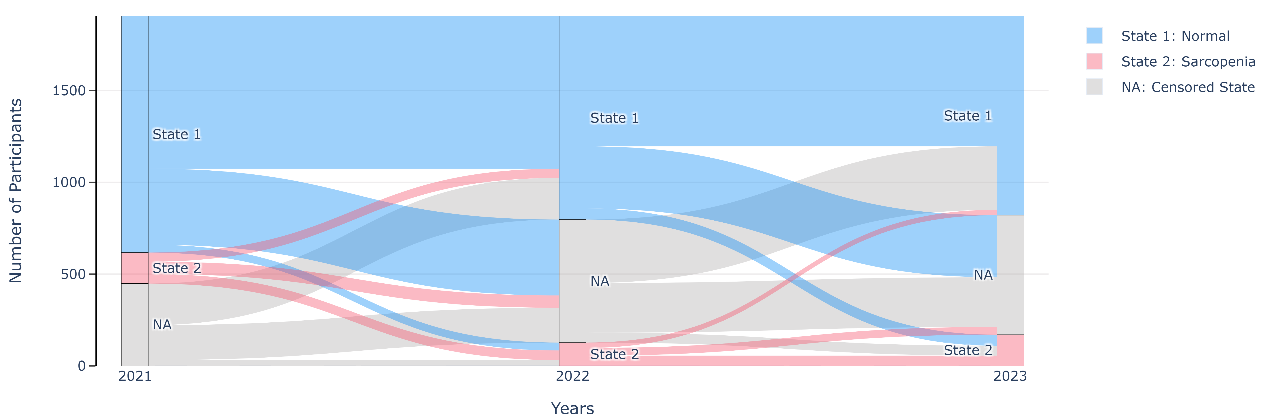
**

Figure S1. The Sankey diagram visualizing transitions between normal and sarcopenia during 2021-2023.

**Note:** The censored state consisted of death, loss to follow-up and unavailable data.
